# Introduction of specialized heart failure nurses in primary care and its impact on readmissions

**DOI:** 10.1017/S1463423622000676

**Published:** 2022-12-09

**Authors:** Robert S. Kristiansson, Richard Ssegonja, Alina Ropponen, Anna Olsson, Filipa Sampaio

**Affiliations:** 1 Department of Public Health and Caring Sciences, Uppsala University, Uppsala, Sweden; 2 Department of Medical Sciences, Respiratory-, Allergy- and Sleep Medicine Research Unit, Uppsala University, Uppsala, Sweden; 3 Primary Care Research Centre, Region Uppsala, Uppsala, Sweden

**Keywords:** care programmes, heart failure, heart failure nurse, quality improvement, readmission

## Abstract

**Background:**

Heart failure (HF) has a 2% prevalence in the population and is a major cause of morbidity and mortality. Multiple efforts have been made worldwide to improve quality of care and decrease unplanned readmissions for HF patients, one of which has been the introduction of specialist HF nurses (HFN) in primary health care. The present evidence on the benefits of HFN is contradicting. This study aims to evaluate the impact of a quality improvement intervention, availability of a HFN in Swedish primary care, on hospital readmissions.

**Methods:**

All patients over the age of 65 with a HF diagnosis and with complete information on availability of a HFN were included in this retrospective register-based study. Using propensity score matching (PSM) techniques, two comparable groups of 128 patients each were created according to the exposure status, availability or no availability of a HFN. The rate of readmission was compared between the groups.

**Results:**

Using PSM, 256 patients were matched, 128 in the HFN group and 128 in the no-HFN group. A total of 50% and 46.09% of patients in the HFN and no-HFN groups were readmitted, respectively. Mean number of readmissions per patient was 1.19 (SD 0.61) in the HFN group and 1.10 (SD 0.44) in the no-HFN group. Patients in the HFN had 17.6% higher odds of being readmitted during the study period, OR: 1.176 (CI: 0.716–1.932), and 3.8% lower odds of being readmitted within 30 days, OR: 0.962 (CI: 0.528–1.750).

**Conclusions:**

Availability of a HFN in primary care was not significantly associated with reduced readmissions for the patients included in this study. Further investigations are warranted looking at the impacts of availability and access to a HFN in primary care on readmissions and other patient outcomes.

## Background

Heart failure (HF) is a major cause of death in developed countries, especially among the elderly (Bui *et al*., 2011). HF is associated with a growing healthcare burden (Groenewegen *et al.*, 2020) The overall prevalence of HF in 13 European countries in 2019 was 1.7% (Seferović *et al.*, 2021). A Swedish population study from 2013 (Zarrinkoub *et al.*, 2013), with a sample of 2.1 million people, estimated that 0.5% of women and 1% of men aged 50–59 years and 18% of women and 21% of men aged 80–89 have HF. This poses a major challenge to health care as HF is a leading primary diagnosis for hospitalization, with more than one million discharged patients in the United States, in 2010 (Ziaeian and Fonarow, 2016). The same relative levels are reported for Europe (Groenewegen *et al.*, 2020). One important and well-accepted indicator for good care processes for patients with chronic conditions is the rate of readmissions. It is used in most healthcare systems, and it is a compound indicator of too early discharge, insufficient planning and follow-up. It is a measure of how well coordination of care is performed (SALAR, no date). Patients with HF are readmitted to a large extent. A study performed in 2009–2012 including patients from 4500 American hospitals reported a 23% risk of readmission within 30 days for HF patients, after being discharged to their homes (Suter *et al.*, 2014).

HF is one of a number of medical conditions, both acute and chronic, that have been considered as ambulatory care sensitive conditions (ACSCs), which should be treated outside the hospital, in an ambulatory care setting (Socialstyrelsen, 2014). As HF can be treated with good medical results in an ambulatory setting, preventing potentially harmful and expensive hospitalization and readmissions is an increasing priority for healthcare leaders and clinicians (Imison *et al*., 2017). However, based on medical population registers, there has been no significant change in the levels of readmissions for ACSCs, in Sweden, over the period 2010–2019 (Socialstyrelsen, 2021).

To support the transition of HF care from hospitals to primary care or other outpatient care, multiple initiatives or quality improvement (QI) efforts have been implemented and evaluated worldwide (Imison *et al*., 2017). Improvement efforts for HF care can be divided into medical treatment, adherence to HF regimes, and improvement of the care process by either focusing on transitions between care levels or direct patient support. Efforts to improve adherence to medical regimens have been successful. Data from 2007 to 2009 showed a 50% adherence to medical regimes (Zhang *et al.*, 2013), and a systematic review of improvement of adherence to medical regimes from 2017 pointed out that five out of the eight (63%) included studies showed improvement in adherence (Andrews *et al*., 2017).

HF patients are typically treated by more than one healthcare provider, and more than 40% of HF patients have five comorbidities or more (Ahluwalia *et al.*, 2011; Stewart *et al*., 2016), making communication between the patient, the next of kin and the providers essential. Considering the complexity of HF care, a representative model of delivering integrated care would involve multiple specialists (Brunner-La Rocca *et al.*, 2015). The challenges for patients with HF are therefore the cooperation between different caregivers, as well as managing the transitions between them. Evidence-based guidelines for the diagnosis, treatment and management of HF aid effective and safe practice (Ponikowski *et al.*, 2016), and there is a standard of care that HF patients should expect even though the results vary (Timmis *et al.*, 2017).

QI interventions aim to enhance the efficiency and effectiveness of health programmes, services or organizations (Batalden and Davidoff, 2007). A systematic review of 25 studies from high-income countries, in 2004–2016, showed a decline in readmission rates for HF patients (Nuckols *et al.*, 2017).

One example of QI interventions is the introduction of specialist HF nurses (HFN), who, in an outpatient setting, follow up patients, optimize therapy, coordinate care and educate patients about self-care. One important role for the HFN is to help the patient to improve self-management. A systematic review of studies on the self-management of HF (Toback and Clark, 2017), covering 1999–2016, shows that improved self-management increases compliance, promotes patient quality-of-life, advances clinical outcomes, reduces hospital readmission and decreases hospitalization costs. Conversely, a more recent Swedish study (Gianluigi *et al.*, 2019), based on the national quality register Swedish Heart Failure Registry, including 40 992 hospital discharges from the whole country during 2000–2012, points out that 39% of patients are referred to a HFN in a primary care setting, and that having a HFN is associated with decreased mortality but not decreased readmission.

On the basis of studies supporting the benefits of HFN in primary care on patient outcomes, Region Uppsala, in central Sweden, initiated in 2016 the implementation of a QI multicomponent programme – The Heart Failure Program (HFP) – to improve the care for patients with HF on equal terms. As HF patients receive treatment from multiple caregivers, including the hospital, primary care and home care, one of the components of the HFP was the introduction of specialist HFN in primary care. The HFN are employed at the primary healthcare centres (PHC), and they integrate a team with a medical doctor, who is responsible for the treatment and follow-up of patients with a HF diagnosis at that PHC. Normally, a patient discharged from the hospital will be followed up at their local PHC, and by both a medical doctor and a HFN, who is their primary contact.

The HFN, as a component of the larger HFP QI effort, has not been evaluated in the context of Swedish primary care in a natural setting. The importance of evaluating QI improvement efforts in health care is indisputable, given its potential to contribute evidence to the benefits of current practice and to further improvement of existing QI interventions. This study aims to evaluate the impact of a QI intervention, availability of a specialist HFN in primary care, on readmissions, in a naturalistic setting.

## Methods

### Study design and data sources

We adopted a retrospective register-based study design using individual patient data from Region Uppsala’s Electronic Patient Medical Records for the period between January 2019 and December 2019. This database collects routine medical patient data on inpatient, outpatient care and medication for about 383 000 inhabitants. Pseudonymized data on patient demographic characteristics, hospital admission and discharge, as well as diagnostic and procedural codes were accessed after ethical approval by the Swedish Ethical Review Authority (Ref: 2019-03208). The Ethical Board did not ask for written or oral consent from every individual, as this was a retrospective study of all patients with HF, based on hospital admission data.

### Study population

**Figure 1.**
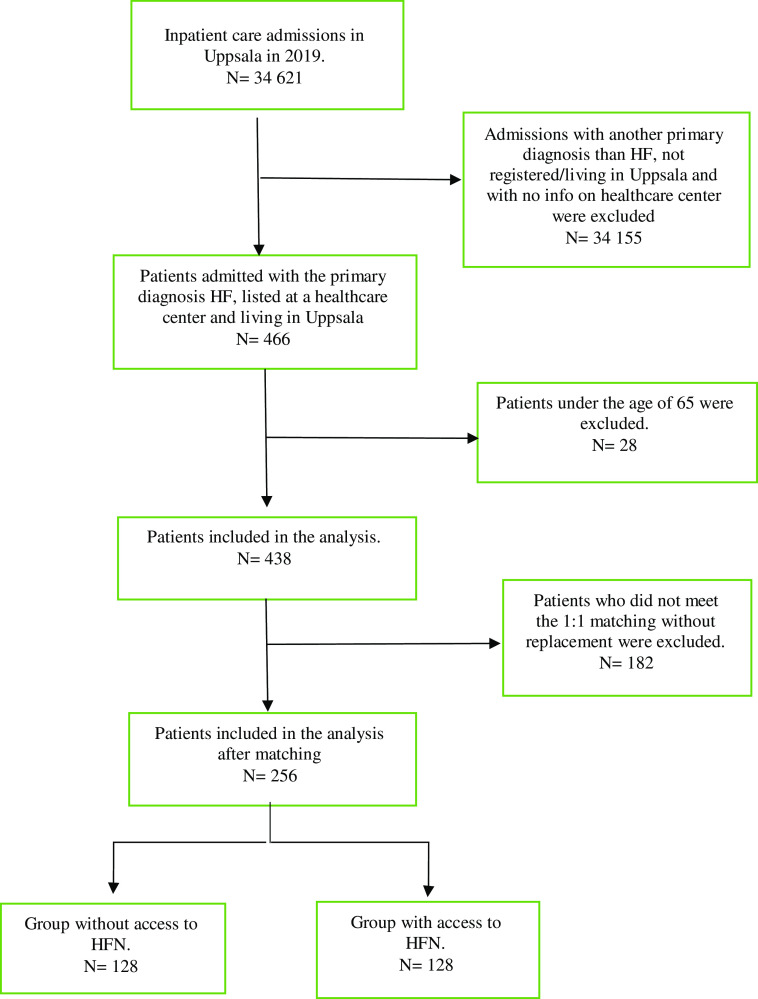
Flow chart of the sample selection process

The study sample was restricted to patients aged 65 and older, hospitalized with a primary diagnosis of HF, between January 2019 and December 2019, and registered at a PHC in Region Uppsala. HF diagnosis was identified based on the International Classification of Diseases – 10^th^ Revision (ICD-10) codes: I500, I501 and I509. The dataset included information on patients’ age, sex, comorbidities, PHC, date of hospitalization, date of discharge, date of readmission and number of days to readmission. Data on mortality were not provided. See Figure 1 for the study sample selection.

### Outcomes and exposure variable

The primary outcomes of interest were readmissions during the study period and readmissions within 30 days of discharge and included all types of readmissions whether planned or unplanned. Planned readmissions are very rare in the Swedish system and count for a small fraction of the total number. The exposure variable was a binary variable indicating whether the patient was listed in a PHC with a specialist HFN or without a specialist HFN. Based on exposure status, patients were categorized into two groups, HFN versus no-HFN. Information on which PHC had HFN as part of their care team was provided by co-author AO in her role as a HFN coordinator.

### Confounders

A set of observed patient-level variables deemed to have the ability to confound the relationship between exposure and outcomes were used in the matching procedure and also adjusted for in the regression analysis (Sherer *et al.*, 2016; Marques *et al.*, 2017; Kwok *et al.*, 2018; Lam *et al.*, 2019). These variables included age, sex and number of comorbidities. Sex was treated as a categorical variable, male and female, and age was treated as a continuous variable. Comorbidities were treated as a categorical variable, where four groups (0–2, 3–5, 6–8 and >09) denoting the numerical range of comorbidities were created from the original continuous variable.

### Statistical analyses

#### Descriptive statistics

Descriptive statistics before and after matching were carried out for the primary outcomes, exposure and confounding variables of the study population. Normality was examined using the Kolmogorov–Smirnov statistical test for normality and graphically using histograms. Means and standard deviations were used to describe the continuous variables, while counts and percentages were used for the categorical variables. Differences between groups were examined using t-tests or Mann–Whitney tests based on whether variables were normally distributed or not. Chi-square tests were used for categorical variables. Fischer’s exact test was used for situations where there were less than five participants per cell, and thus a Chi-square test could not be used. No missing data were present.

#### Matching

Patients in the HFN group were matched with patients in the non-HFN group based on the available observable confounders: age, sex and comorbidities, using propensity score matching (PSM) methods (Zhang *et al.*, 2019). The PSM enables making the groups comparable by pairing individuals in the HFN group with individuals in the non-HFN group, based on observable characteristics. Making the groups comparable allows us to examine the relationship between availability of a HFN and readmissions with minimal effect on the observed confounders. A 1:1 matching without replacement, with a k-nearest neighbour approach, was employed. A 1:1 matching without replacement means that for each individual in the HFN group with a certain age, sex and number of comorbidities, one similar individual was found in the non-HFN group, where each individual in the non-HFN group could only serve as a match once. A k-nearest neighbour approach finds a match with the closest propensity score (a weight between 0 and 1 assigned to each participant). Matching without replacement and a k:1-nearest neighbour approach are a good choice for its simplicity and good performance in situations where there are more controls than “treated” individuals, such as the present study (Stuart, 2010). Standardized mean differences (SMD) and percentage bias were used to examine balance in covariates between groups after PSM (Austin, 2011; Heinze and Jüni, 2011; Ssegonja *et al.*, 2019; Zhang *et al.*, 2019). *P*-values over 0.05 and SMD less than 5% indicated good matching (Ssegonja *et al.*, 2019). The success of the matching was also examined graphically using the psgraph command, by examining the areas of overlapping between the exposed and non-exposed groups. Matching with replacement was also implemented as an extra analysis, resulting in a smaller sample and with no change in final results. The PSM was performed in STATA version 15 (StataCorp) using the psmatch2 command (Leuven and Sianesi, 2018).

#### Exposure effect analysis

To examine the relationship between availability of a HFN and readmissions, a binary logistic regression analysis was done. The confounding variables age, sex and number of comorbidities, used in the PSM, were also controlled for in the regression analysis to enhance precision given the difficulties of obtaining perfect balance in matching procedures. Results from the logistic regression were expressed as odds ratios, and significance was set at 0.05. The regression analyses were performed in SPSS 26.

## Results

### Study population

In total, 438 (48.40% female) patients hospitalized with the primary diagnosis of HF were eligible for the study. The mean age was 81.76 years (SD 7.63), mean number of comorbidities was 5.85 (SD 2.65) and 51.61% had six or more comorbidities. The HFN group included 130 patients and the no-HFN group included 308 patients. Before matching, there was a statistically significant difference in age between the HFN group and the no-HFN group. All other differences with regard to the observed variables were not statistically significant (Table 1).

**Table 1.** Sample characteristics before matching

	Total	HFN	No-HFN	
Variable	*N* (%)	*N* (%)	*N* (%)	*P*-value
Patients	438 (100)	130 (29.68)	308 (70.32)	
Age, mean (SD)	81.76 (7.63)	83.65 (7,56)	80.96 (7.53)	0.001*
Sex				0.687
Female	212 (48.40)	61 (46.92)	151 (49.03)	
Male	226 (51.60)	69 (53.08)	157 (50.97)	
Number of comorbidities, mean (SD)	5.85 (2.65)	5.82 (2.47)	5.86 (2.73)	0.957
Number of comorbidities				0.441
0–2	39 (8.90)	11 (8.46)	28 (9.09)	
3–5	173 (39.50)	57 (43.85)	116 (37.66)	
6–8	155 (35.39)	39 (30.00)	116 (37.66)	
≥ 9	71 (16.21)	23 (17.69)	48 (15.58)	
Admitted from				0.457
Home	410 (93.61)	123 (94.62)	287 (93.18)	
Unit within Region Uppsala	22 (5.02)	7 (5.38)	15 (4.87)	
Unit outside Region Uppsala	2 (0.46)	0	2 (0.65)	
Elderly care home	4 (0.91)	0	4 (1.30)	
Discharged to				0.889
Home	394 (89.95)	115 (88.46)	279 (90.58)	
Unit within Region Uppsala	4 (0.91)	1 (0.77)	3 (0.97)	
Unit outside Region Uppsala	3 (0.68)	1 (0.77)	2 (0.65)	
Elderly care home	37 (8.45)	13 (10.00)	24 (7.79)	
Number of admissions per patient, mean (SD)	1.14 (0.48)	1.15 (0.47)	1.13 (0.50)	0.398
Number of patients being readmitted	219 (50.00)	65 (29.68)	154 (70.32)	1.000
Acute admissions (% of readmissions)	165 (75.34)	49 (75.38)	116 (75.32)	0.992
Number of readmissions per patient, mean (SD)	1.18 (0.58)	1.18 (0.61)	1.18 (0.57)	0.999
Heart failure as main diagnosis at readmission (% of readmissions)	74 (33.79)	17 (26.15)	57 (37.01)	0.121
Readmissions within 30 days (% of readmissions)	100 (45.67)	28 (43.08%)	72 (46.75%)	0.675
Acute admissions (% of readmissions within 30 days)	77 (77.00)	22 (78.57)	55 (76.39)	0.816
Readmissions beyond 30 days (% of readmissions)	131 (59.82)	40 (61.54%)	91 (59.10%)	0.798
Number of days to readmission, mean (SD) (only for readmitted)	69.73 (72.14)	70.45 (71.26)	69.43 (72.74)	0.929
Mortality	4 (0.009%)	0 (0%)	4 (100%)	–

SD = standard deviation; HFN = heart failure nurse.

*Statistically significant.

In the PSM, we were able to match 256 patients, 128 in the HFN group and 128 in the no-HFN group, whereas the remaining patients in the HFN group had no matching peer. The mean age was 83.45 years (SD 7.44) in the HFN group and 83.37 years (SD 7.26) in the no-HFN group. The mean number of comorbidities was 5.85 (SD 2.46) in the HFN group and 5.81 (SD 2.57) in the no-HFN group. A total of 50% and 46.09% of patients in the HFN and no-HFN groups were readmitted, respectively. Mean number of readmissions per patient was 1.19 (SD 0.61) in the HFN group and 1.10 (SD 0.44) in the no-HFN group. Of these, 43.75 % in the HFN group and 49.15% in the no-HFN group were within 30 days, with the majority registered as acute (78.57% vs 72.41%, HFN and no-HFN group, respectively). Number of days to readmission were 67.97 (SD 68.94) in the HFN group and 61.34 (SD 67.64) in the no-HFN group.

The matched sample was balanced in terms of all observed variables and confounders, and there were no statistically significant differences between groups after matching (Table 2).

**Table 2.** Sample characteristics for the matched sample, stratified by availability of a specialist HFN

	HFN	No-HFN	
Variable	N (%)	N (%)	*P*-value
Patients	128	128	
Age, mean (SD)	83.45 (7.44)	83.37 (7.26)	0.954
Sex			0.707
Female	61 (47.66 %)	58 (45.31%)	
Male	67 (52.34%)	70 (54.69%)	
Number of comorbidities, mean (SD)	5.85 (2.46)	5.81 (2.57)	0.816
Number of comorbidities			0.584
0–2	10 (7.81%)	9 (7.03%)	
3–5	56 (43.75%)	52 (40.63%)	
6–8	39 (30.47%)	49 (38.28%)	
≥ 9	23 (17.97%)	18 (14.06%)	
Admitted from			0.525
Home	121 (94.53%)	118 (92.19%)	
Unit within Region Uppsala	7 (5.47%)	9 (7.03%)	
Unit outside Region Uppsala	0 (0%)	0 (0%)	
Elderly care home	0 (0%)	1 (0.78%)	
Discharged to			0.946
Home	113 (88.28%)	113 (88.28%)	
Unit within Region Uppsala	1 (0.78%)	2 (1.56%)	
Unit outside Region Uppsala	1 (0.78%)	1 (0.78%)	
Elderly care home	13 (10.16%)	12 (9.38%)	
Number of admissions per patient, mean (SD)	1.13 (0.51)	1.11 (0.38)	0.874
Number of patients being readmitted	64 (50%)	59 (46.09%)	0.532
Acute admissions	49 (76.56%)	45 (76.27%)	0.970
Number of readmissions per patient, mean (SD)	1.19 (0.61)	1.10 (0.44)	0.288
Heart failure as main diagnosis at readmission (% of readmissions)	17 (26.56%)	19 (32.20%)	0.492
Readmissions within 30 days (% of readmissions)	28 (43.75%)	29 (49.15%)	0.881
Acute admissions	22 (78.57%)	21 (72.41%)	0.589
Readmissions beyond 30 days (% of readmissions)	39 (60.94%)	32 (54.24%)	0.328
Number of days to readmission, mean (SD) (only for readmitted)	67.97 (68.94)	61.34 (67.64)	0.597
Mortality	0 (0%)	2 (100%)	–

SD = standard deviation; HFN = heart failure nurse.

**Table 3.** Results of binary logistic regression analysis

Outcome		B	Sig.	Exp(B)	95% CI	
					Lower	Higher
Readmitted	Unadjusted	0.157	0.532	1.169	0.716	1.910
	Adjusted*	0.162	0.521	1.176	0.716	1.932
Readmitted within 30 days	Unadjusted	**−0.045**	**0.881**	**0.956**	**0.530**	**1.722**
	Adjusted*	**−0.039**	**0.898**	**0.962**	**0.528**	**1.750**
Readmitted within 30 days and acute	Unadjusted	0.074	0.832	1.076	0.546	2.123
	Adjusted*	0.087	0.806	1.091	0.547	2.176

CI = confidence interval.

* Adjusted for sex, age and number of comorbidities. Significance of bold values was set at 0.05.

When examining the relationship between availability of a HFN and readmissions using a binary logistic regression analysis (Table 3), in all adjusted models, we found non-statistically significant larger odds (17.6%) for patients in the HFN group of being readmitted (including both readmissions within and beyond 30 days). For the same group of patients in the HFN group, we also observed lower odds (3.8%) of being readmitted within 30 days, although these results were not statistically significant. For the outcome acute readmissions within 30 days, patients in the HFN group showed non-statistically significant larger odds (0.9%) than patients in the non HFN group.

## Discussion

In this study, using PSM and regression analysis while controlling for observed confounders, no significant effect was found of the impact of availability of a HFN on overall readmissions or on readmissions within 30 days for patients with HF. We note a numerical difference in form of an increase in mean total readmissions in the HFN group and a decrease in mean readmission within 30 days, though non-significant. This study was done in an early stage of a QI initiative aiming at improving HF care. There is a possibility that the cohort of HF patients had urgent healthcare needs as identified by HFN and therefore accurately sent to the hospital for treatment. This could be one possible explanation to the larger readmission rates for these patients.

Our findings are not in concordance with the results reported in the systematic review of 25 trials showing lower readmission rates related to the implementation of multicomponent QI interventions (Nuckols *et al.*, 2017). A possible reason is that studies in the literature reporting positive findings are often from controlled environments where QI is only a small part of a larger research project. The results from the present study are, however, similar to the results reported by Gianluigi *et al.* (2019), including 40 992 hospital discharges in Sweden, from 2000 to 2012. Gianluigi *et al.* reported that referral to a HFN clinic in primary care did not lead to a reduction in readmissions.

This study was performed in a naturalistic setting where a HFN had been introduced during the last 2–3 years in only some of the PHC. The available data, including all patients with HF diagnosis over 65 years of age, have given the authors the opportunity to better understand the effects of the implementation of a QI intervention, in particular of the availability of a specialist HFN in Swedish primary care, on readmission. The available data also show that only approximately 30% of the patients in this study had availability of a HFN at their PHC, which reveals that the implementation has not yet been completed.

The HFN in our study had the important role to help the patient with improved self-management, which has, in a previously published systematic review by Toback *et al.* (Toback and Clark, 2017), been shown to reduce hospital readmissions.

The current study had many strengths. It investigates a unique QI intervention in a Swedish setting, which is valuable, as results from other settings cannot be directly transferred to the Swedish healthcare setting due to existing structural differences, including for instance where and how HF patients are managed (Wang *et al.*, 2013). The analyses were based on real-life data from patient electronic medical records, which reflects routine praxis and overcomes the constraints of controlled studies, including non-representative study populations, missing data and limitations of self-reported data. Second, readmission rate is an accepted healthcare quality indicator used in multiple studies and evaluations; hence, one is able to compare results across studies. Third, the use of PSM was used to attempt to create comparable groups and reduce uncertainty in the study results.

The current study also has limitations. We used the best available register data to investigate whether availability of HFN had any impact on readmissions; however, we were not able to ascertain whether patients listed at PHC with a HFN did in fact have actual contact with and were treated by one. A more detailed investigation on an individual level could reveal what kind of contact there had been with the HFN, and thus actual exposure to the evaluated intervention. A partnership between the HFN and a medical doctor could have an impact on readmission, but that data were not available within this study. Additionally, data limitations hampered the possibilities of gathering more detailed information on the competencies and practices of each individual HFN to understand if there were variations in readmission rates between different HFN. Another limitation is that there was no information on the severity of HF for each patient. It is likely that patients with more severe HF, that is, a higher New York Heart Association (NYHA) functional classification stage, are more likely to be readmitted regardless of whether they had availability to a HFN or not. It is also possible that patients with higher NYHA class were more likely to be assigned to a HFN in practices where these were available, thus introducing bias. Further, it was not possible to get information on the severity of comorbidities, which is thought to explain some of the variation in the readmissions. In the current study, categories were grouped based on numbers of comorbidities and not on severity. Data on socioeconomic status as well as mortality were unavailable which would have been an informative outcome to study. Lastly, no evaluation protocol was integrated within the system designed to monitor and evaluate the impacts of the QI initiative. This limited the possibility for the PHC management to assess the effects of the programme.

For improved patient outcomes and organizational performance, integrated systems for monitoring and evaluation need to be in place to collect data that enable the assessment of the impacts of QI initiatives that introduce new competencies or capacities, such as the HFN, on both individual and system levels.

## Conclusions

We conclude that the availability of a HFN in primary care was not significantly associated with a reduction in readmissions for the patients included in this study. Further investigations are warranted looking at the impacts of availability and access to a HFN in primary care on readmissions and other patient outcomes, specifically assessing which components of this intervention are potentially effective.
